# Enhancing the Anticancer Activity of *Antrodia cinnamomea* in Hepatocellular Carcinoma Cells via Cocultivation With Ginger: The Impact on Cancer Cell Survival Pathways

**DOI:** 10.3389/fphar.2018.00780

**Published:** 2018-07-18

**Authors:** San-Yuan Chen, Ying-Ray Lee, Ming-Chia Hsieh, Hany A. Omar, Yen-Ni Teng, Ching-Yen Lin, Jui-Hsiang Hung

**Affiliations:** ^1^Department of Chinese Medicine, Ditmanson Medical Foundation Chia-Yi Christian Hospital, Chiayi, Taiwan; ^2^Institute of Molecular Biology, National Chung Cheng University, Chiayi, Taiwan; ^3^Department of Medical Research, Ditmanson Medical Foundation Chia-Yi Christian Hospital, Chiayi, Taiwan; ^4^Department of Internal Medicine, Changhua Christian Hospital, Changhua, Taiwan; ^5^Sharjah Institute for Medical Research and College of Pharmacy, University of Sharjah, Sharjah, United Arab Emirates; ^6^Department of Pharmacology, Faculty of Pharmacy, Beni-Suef University, Beni Suef, Egypt; ^7^Department of Biological Sciences and Technology, National University of Tainan, Tainan, Taiwan; ^8^Drug Discovery and Development Center, Chia Nan University of Pharmacy and Science, Tainan, Taiwan; ^9^Department of Biotechnology, Chia Nan University of Pharmacy and Science, Tainan, Taiwan

**Keywords:** *Antrodia cinnamomea*, ginger, hepatocellular carcinoma, apoptosis, cell cycle, MAPK, adjuvant therapy

## Abstract

*Antrodia cinnamomea* (*AC*) is a medicinal fungal species that has been widely used traditionally in Taiwan for the treatment of diverse health-related conditions including cancer. It possesses potent anti-inflammatory and antioxidant properties in addition to its ability to promote cancer cell death in several human tumors. Our aim was to improve the anticancer activity of *AC* in hepatocellular carcinoma (HCC) through its cocultivation with ginger aiming at tuning the active ingredients. HCC cell lines, Huh-7 and HepG2 were used to study the *in vitro* anticancer activity of the ethanolic extracts of *AC* (EAC) alone or after the cocultivation in presence of ginger (EACG). The results indicated that the cocultivation of *AC* with ginger significantly induced the production of important triterpenoids and EACG was significantly more potent than EAC in targeting HCC cell lines. EACG effectively inhibited cancer cells growth via the induction of cell cycle arrest at G2/M phase and induction of apoptosis in Huh-7 and HepG2 cells as indicated by MTT assay, cell cycle analysis, Annexin V assay, and the activation of caspase-3. In addition, EACG modulated cyclin proteins expression and mitogen-activated protein kinase (MAPK) signaling pathways in favor of the inhibition of cancer cell survival. Taken together, the current study highlights an evidence that EACG is superior to EAC in targeting cancer cell survival and inducing apoptotic cell death in HCC. These findings support that EACG formula can serve as a potential candidate for HCC adjuvant therapy.

## Introduction

Hepatocellular carcinoma is a common tumor influencing more than one million individuals worldwide every year (Mittal and El-Serag, 2013). HCC is the fifth most frequent cancer worldwide and the second most common cause of cancer death in the world (Omar et al., 2016b; Zhu et al., 2016). HCC occurs both sporadically and in relation to transgenic oncogenes (Jain et al., 2010), viral infection (Wang et al., 2003; Hung et al., 2004), environmental exposure (Hung et al., 2015; Rieswijk et al., 2016), extensive alcohol intake (Thorgeirsson and Grisham, 2002), and other causes of hepatic cirrhosis. HCC usually has poor prognosis because of the resistance to ordinary chemotherapy and constrained adequacy of radiotherapy (Avila et al., 2006; Omar et al., 2014). Therefore, there is a current need for novel compounds or natural products to tackle HCC without those drawbacks.

*Antrodia cinnamomea* is a valuable and unique edible fungus originating in Taiwan. AC has been utilized by native clans for quite a long time to treat nourishment inebriation and to enhance liver functions (Wen et al., 2011; Peng et al., 2017). It was cultivated using four major culture techniques including liquid fermentation, solid support culture, cut wood culture, and dish culture. The crude extracts of AC by ethanol extraction have been commonly used in the Taiwanese market as health food products. Many biological activities of AC have been demonstrated such as anti-inflammatory, cytotoxic and hepatoprotective properties. For anti-inflammatory activity, many compounds from AC have been reported. For example, antrodin D was isolated from the fruiting bodies of AC (Chien et al., 2008). In addition, antrocinnamomin A, an active component of AC mycelia (ACM), displayed a significant NO inhibitory activity in LPS-stimulated RAW264.7 macrophages (Wu et al., 2008). Considering the cytotoxic activity, it was reported that camphorataimide B displayed a potent anticancer activity in human breast cancer, leukemia cells, and human lung cancer cells (Lin et al., 2012). For hepatoprotective activity, maleic and succinic acid derivatives from the AC mycelia were involved in inhibition of HCV protease (Phuong do et al., 2009). In addition, some of the *A. cinnamomea* extract components such as methyl antcinate A, antcin B, and antcin K were able to induce apoptotic cell death in HCC (Hsieh et al., 2010, 2011; Huang et al., 2015; Lai et al., 2016).

Ginger, the rhizome of *Zingiber officinale*, is one of the most widely used traditional medicinal herbs which possess antioxidant, anti-inflammatory and anticancer properties (Park et al., 1998; Pan et al., 2008a; Wu et al., 2015). Many chemical compounds were identified in ginger rhizome, such as gingerol, shogaol, paradols, and gingerdiols. Diverse biological activities of ginger extract active compounds have been reported. For example, 6-gingerol suppresses T cell activation and proliferation, which results in the prevention or alleviation of allergic rhinitis symptoms (Kawamoto et al., 2016). The activation of the PPARδ pathway by ginger extract reduced diet-induced obesity and 6-Shogaol and 6-gingerol might be responsible for the impacts of the dietary ginger on PPARδ signaling (Misawa et al., 2015). In addition, the combination of gelam honey and ginger induced colon cancer cells apoptosis through the modulation of mTOR and Wnt/β-catenin pathways (Wee et al., 2015). Furthermore, the inhibition of breast cancer cells and stem cell-like spheroids by 6-shogaol was through the modulation of the notch signaling pathway (Ray et al., 2015). 6-shogaol has additionally been found to instigate apoptosis in human colorectal carcinoma cells by means of ROS generation, caspases activation and the induction of GADD 153 expression (Pan et al., 2008b). Also, 6-shogaol prompted autophagy in human non-small cell lung cancer A549 cells by repressing the AKT/mTOR pathway (Hung et al., 2009). Our previous study indicated that increased ROS production, ER stress and autophagy were observed in response to 6-shogaol treatment in HCC (Wu et al., 2015). Other ginger components, for example, 10-gingerol, restrains the multiplication and invasion of MDA-MB-231 breast cancer cells through the concealment of Akt and p38 MAPK activity (Joo et al., 2016). Based on these anticancer activities of ginger extract, here we hypothesized that the employment of fresh ginger as a cultivation medium for *AC* would improve its anticancer activities. The outcome of the current study may serve as a basis to develop a novel formula of EAC extract to be used in both cancer prevention and treatment.

## Materials and Methods

### Cell Culture

HepG2 and Huh-7 cell lines were by provided Dr. M.D. Lai at National Cheng Kung University. Cells were incubated at 37°C in a 5% CO_2_ incubator with DMEM containing 10% fetal bovine serum.

### Chemicals and Reagents

ECL detection system for Western blot was from Millipore (Billerica, MA, United States). Anti-Akt, p-Thr308-Akt, β-actin were obtained from Santa Cruz Biotechnology (Dallas, TX, United States). Anti-p38, ERK, JNK, p-p38, p-ERK and p-JNK, cyclin B1, cyclin D1, cyclin A, cyclin H, cyclin E1 antibodies were purchased from Cell Signaling (Beverly, MA, United States). The secondary antibodies, anti-rabbit IgG-horseradish peroxidase and rabbit anti-mouse IgG-horseradish peroxidase, were purchased from Jackson ImmunoResearch (West Grove, PA, United States). Crystal violet, acetonitrile, Dimethyl sulfoxide, methanol (HPLC grade), isopropanol, *N,N,N*′,*N*′-Tetramethylethylenediamine, glycine, sodium lauryl sulfate, ammonium persulfate, and MTT were purchased from Sigma-Aldrich (St. Louis, MO, United States). Tris-HCl and Acrylamide/Bis-acrylamide (30%/0.8% w/v) were obtained from MDBio (Taipei, Taiwan). The water for HPLC analysis was purified using a Milli-Q water purification system (Millipore, Burlington, MA, United States).

### Cell Viability Assay

Cell viability for EAC and EACG treatment was evaluated with MTT assay in Huh-7 and HepG2 cells as mentioned before (Omar et al., 2013). The MTT assay correlates the cellular metabolic activity with NAD(P)H-dependent cellular oxidoreductase enzymes activities which can reflect the number of viable cells present. Briefly, Huh-7 or HepG2 cells (7 × 10^3^/well) were incubated in 96-well culture plates. Cells were treated with EAC, EACG or EACF at different concentrations. After 48 h incubation, the EAC or EACG-containing medium was replaced with 0.5 mg/mL MTT-culture medium (100 μl/well). The 96-well plate was placed in CO_2_ incubator at 37°C for 4 h. Then MTT-containing media were removed and DMSO (100 μl/well) was used to dissolve blue formazan crystals. The developed color was measured at 570 nm using an ELISA reader.

### Preparation of *A. cinnamomea* and Ginger Extracts

*Antrodia cinnamomea* was purchased from Bioresource Collection and Research Center (Hsinchu, Taiwan; strain number: BCRC 35398) and was incubated in M25 medium (2% Glucose, 2% Malt extract, 0.1% peptone and 2% agar) with or without 1% ginger (weight/volume) at 25°C for 50 days. Since the water extract of ginger exhibits antifungal activity at concentrations over 2.5%, which may inhibit the growth of *AC*, we used only 1% of the ginger for the co-cultivation based on a pilot study for the selection of optimal ginger concentration (Touba et al., 2012). The *AC* frozen dried plates, fruiting body and ginger frozen dried plates were then incubated with 95 and 75% ethanol for 3 days, and the total crude extracts were concentrated by rotary evaporator, and the dried extracts were then dissolved in DMSO. The EAC, EACG, EACF and ethanolic extracts of ginger (EG) stock solutions were prepared in DMSO at concentration of 50 mg/ml and stored at -20°C. For each experiment, the extracts were freshly prepared with a final DMSO concentration of 0.1%. Control treatments received equivalent amount of DMSO (0.1% v/v).

### HPLC and LC-MS/MS Analysis of *A. cinnamomea* Extracts

The analysis of the EAC, EACG and EACF extracts was performed on a liquid chromatography system (Hitachi, L2130, Tokyo, Japan). An auto-sampler (Chromaster 5210) with a vacuum degasser, 20 μL loop, diode-array detector (L-7455), and quaternary pump (Chromaster 5110) were equipped in the system. A Security Guard C18(ODS) precolumn (Phenomenex Inc., Torrance, CA, United States) and Luna C18(2) reversed-phase analysis column have been used for components in extracts during separation. During gradient elution, solvent A (0.1% formic acid, FA in water) and solvent B (acetonitrile with 0.1% FA) were served as the mobile phase at a flow rate of 0.2 mL/min. Peak areas for the main ten compounds present in *A. cinnamomea* extracts were determined at 270 nm. For LC-MS/MS analysis in EAC, EACG, and EACF extracts, an Agilent 6420 Triple Quadrupole Mass Spectrometer and Mass Hunter software (version: B.01.04; Agilent Technologies, Santa Clara, CA, United States) were used for analysis. During TAFC separation, Acquity BEH reverse phase C18 column (1.7 μm, 2.1 mm × 50 mm) was used for analysis. The composition of the mobile phase A and the mobile phase B is: 0.1% formic acid in water and 0.1% formic acid in acetonitrile. The tandem quadrupole mass spectrometer with the positive electrospray ionization (ESI+) mode (0.50 kV capillary voltage, nitrogen gas and 400°C) was used for injection of eluted TAFC. The results were obtained from the multiple reaction monitoring (MRM) mode. The control of the equipment and data acquisition were performed by Waters Empower chromatography software (Waters Limited, Mississauga, ON, Canada).

### Analysis of Cell Cycle

For cell-cycle distribution analysis, 1 × 10^6^ cells were treated with 50 μg/ml, 100 μg/ml, and 150 μg/ml of EAC or EACG for 24 and 48 h (Weng et al., 2010). After incubation, the collected cells were fixed with 5 ml 70% ethanol/PBS at -20° C for 1 h. The fixed cells were washed twice with cold PBS and resuspended with propidium iodide containing RNase A. Cell-cycle distribution was performed using flow cytometry and the results were analyzed by FlowJo software (FLOWJO, LCC, Ashland, OR, United States).

### DAPI Staining

DAPI staining was used to identify the nuclear chromatin morphological changes after EAC or EACG treatment as described before (Omar et al., 2013). Briefly, 7 × 10^5^ cells were plated in 6-cm dishes and incubated at 37°C for 36 h. After treatments with EAC or EACG, the cells were fixed with 3% paraformaldehyde for 15 min. The fixed cells were then stained with DAPI for 2 min, and the cells were washed twice with PBS and pictures were caught utilizing a fluorescence magnifying microscope (Olympus, Tokyo, Japan)

### Colony Formation Assay

To determine the colony formation, HCC cells were seeded in 6-well tissue culture plates and incubated in DMEM culture medium with 10% FBS. Cells were then processed as detailed before (Lee et al., 2014).

### Caspase-3 Activity

To analyze the effect of EAC and EACG on caspase-3 activity as a biomarker of apoptosis, PE active caspase-3 apoptosis kit (BD Pharmingen) was used according to the manufacturer’s protocol. Treated cells were subjected to flow cytometry and caspase-3 activity was analyzed with FlowJo software.

### Annexin V Staining Assay

To determine cell apoptosis with annexin V assay, 7 × 10^5^ HCC cells were seeded in 6-cm cell culture dishes and incubated at 37°C for 16 h. The cells were treated with 50 μg/ml EAC, EACG or vehicle for 48 h. After treatment, the cells were washed twice with cold PBS and stained with propidium iodide and Annexin V for 15 min at room temperature. The apoptotic cell distribution was determined by flow cytometry and analyzed by FlowJo software program.

### Western Blot analysis

For Western blot analysis, the total cell proteins were extracted from treated cells using RIPA lysis buffer. The cell lysates were processed as formerly reported (Arafa el et al., 2014). The βVDF membranes were blotted with primary antibodies against b-actin, NF-kB, Bcl-2, PARP, p38, pp38, JNK, pJNK, ERK, pERK cyclin A, cyclin B1, cyclin D1, and cdc2 antibodies in 1% TBST non-fat milk at 4°C overnight. The membranes were then washed and incubated with corresponding secondary antibodies (anti-rabbit IgG-HRP conjugates or anti-mouse IgG-HRP conjugates) for 1 h at room temperature. Enhanced chemiluminescence kit (GE, Pittsburgh, PA, United States) was used for the detection of blots.

### Statistical Analysis

Statistical comparisons of the cell proliferation assay, annexin V staining assay and caspase-3 assay results were made using the Student’s *t*-test. Differences were considered significant at ^∗^*P* < 0.05, ^∗∗^*P* < 0.01, and ^∗∗∗^*P* < 0.001, respectively.

## Results

### Cultivation of AC and Bioactive Compounds Identification

**FIGURE 1:**
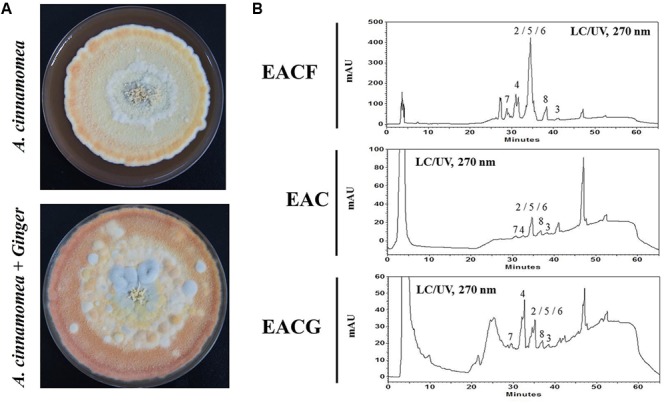
*Antrodia cinnamomea* incubation and LC–UV chromatogram. **(A)** *A. cinnamomea* was incubated with or without 1% ginger for 50 days. The crude extracts were isolated using ethanol. **(B)** HPLC analysis of EACF, EAC and EACG extracts by UV at 270 nm. The peaks numbering corresponds to the natural products shown in **Table 1**.

In order to incubate ginger with *A. cinnamomea*, fresh ginger was grounded into a pulpy state and mixed with M25 culture medium to a final concentration of 1%. The plates were incubated at 25°C for 50 days (**Figure 1A**). The frozen dry plates were then cultivated with 95 and 75% ethanol every 3 days, and the total crude extracts from EAC or EACG were concentrated using a rotary evaporator. HPLC was used to evaluate the bioactive compounds in *A. cinnamomea* fruiting body and *A. cinnamomea* cocultatived with or without 1% ginger. To investigate the different metabolites profile in the samples, HPLC fingerprint of the wild fruiting body ethanolic extract of *A. cinnamomea* (EACG) was used as a standard (**Figure 1B**, upper panel). Various compounds have been identified in EAC, EACG and EACF (**Table 1** and Supplementary Material). The index compounds were: (1) methyl antcinate B, (2) methyl antcinate A, (3) dehydroeburicoic acid, (4) antcin A, (5) antcin B, (6) antcin K, (7) 15α-acetyl dehydrosulphurenic acid, (8) dehydrosulphurenic acid, (9) 3β,15α-dihydroxy-lanosta-7,9(11),24-triene-21-oic acid, (10) zhankuic acid C (Rao et al., 2013). After UV, total ion current (TIC) chromatogram and extracted ion chromatogram (EIC) analysis, compounds 2, 4, 5, and 6 were significantly increased in EACG (**Figure 1B** and Supplementary Material). In addition, the extract compounds were determined by LC/MS/MS. The results indicated that antcin B, dehydrosulphurenic acid, 3β,15α-dihydroxy-lanosta-7,9(11),24-triene-21-oic acid and zhankuic acid C contents were significantly increased upon the cultivation of *A. cinnamomea* with ginger. Interestingly, it was observed that a novel compound with a molecular weight of 470 was also induced in EACG (**Figure 1B**, lower panel).

**Table 1:** The major triterpenoids in different *A. cinnamomea* extracts.

Number	M.W	Compound
1	482	Methyl antcinate B
2	468	Dehydroeburicoic acid/Antcin B/Methyl antcinate A
3	526	15α-acetyl dehydrosulphurenic acid
4	470	3β,15α-dihydroxy-lanosta-7,9(11),24-triene-21-oic acid
5	484	Dehydrosulphurenic acid
6	486	Zhankuic acid C
7	488	Antcin K
8	454	Antcin A

### EACG Has Better Inhibitory Effect on the Cell Viability of HepG2 and Huh-7 Cells Than EAC

**FIGURE 2:**
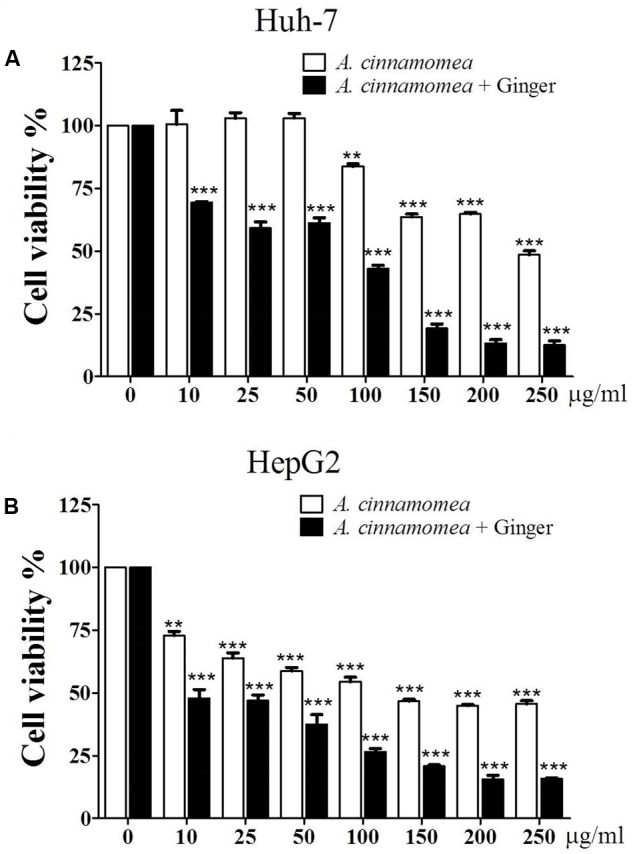
Effects of EAC and EACG on the viability of Huh-7 and HepG2 cells. **(A,B)** MTT assay results showing the inhibition of Huh-7 and HepG2 cell viability by EAC and EACG treatment. Cells were exposed to EAC and EACG in the indicated concentrations in 10% FBS-supplemented DMEM for 48 h. Data represent the mean ± SD (*n* = 6). Significant difference between the control and experimental groups are marked with asterisks (^∗∗^*P* < 0.01; ^∗∗∗^*P* < 0.001).

The effects of EAC or EACG extracts on Huh-7 and HepG2 cells viability were investigated using MTT assay. The cells were incubated with EAC or EACG for 48 h. The results indicated that cell viability of Huh-7 and HepG2 cells was inhibited by EAC and EACG (**Figures 2A,B**). The normality distribution of cell survival results presented in **Figure 2** is available in Supplementary Materials.

However, EACG caused more significant inhibition of cell viability than EAC in both Huh-7 and HepG2 cell lines. For example, at a dose of 50 μg/ml, EACG reduced the cell viability of Huh-7 cells to 60% while EAC showed only a minor effect on Huh-7 cells viability. The half maximal inhibitory concentration (IC_50_) values of EAC and EACG were 245.40 and 50.33 μg/ml in Huh-7 cells and 51.93 and 8.35 μg/ml in HepG2 cells, respectively (**Table 2**).

**Table 2:** The IC_50_ values of EAC and EACG in Huh-7 and HepG2 cells based on MTT cell viability analysis.

Treatment	Huh-7 (IC_50_ μg/ml)	HepG2 (IC_50_ μg/ml)
EAC	245.40 ± 2.45	51.93 ± 2.18
EACG	50.33 ± 3.66	8.35 ± 0.97

*Vales are mean ± SD (*n* = 6). Ethanoic extracts of *Antrodia cinnamomea* (EAC) alone or after the cocultivation in presence of ginger (EACG).*

### EACG Changed Cell Morphology and Decreased Colony Formation in Huh-7 Cells

**FIGURE 3:**
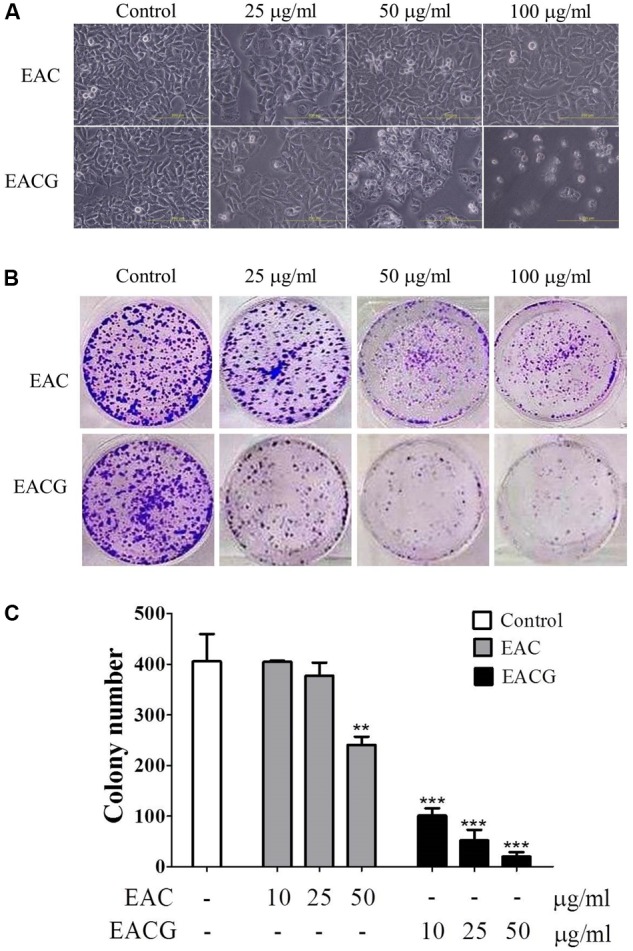
EACG changed cell morphology and colony formation in Huh-7 cells. **(A)** Morphological changes in Huh-7 cells after treatment with EAC or EACG for 48h (photography under phase-contrast magnification). **(B)** Colony formation assay of Huh-7 cells treated with EAC and EACG. Cells were stained by crystal violet after 14 days. **(C)** Bar chart presenting the average number of colonies of three independent experiments. Bars are means ± SD (*n* = 3). Significant difference between the control and experimental groups are marked with asterisks (^∗∗^*P* < 0.01; ^∗∗∗^*P* < 0.001).

To affirm the capacity of EAC or EACG to target HCC, the cells were treated with EAC or EACG and watched for any changes in the cell morphology and colony development ability. For cell morphology, results showed that EACG made morphological changes from flat to round while EAC caused a minor impact on cell morphology (**Figure 3A**). For the effect on colony formation ability of HCC cells, the results indicated that EACG significantly decreased colony formation ability of Huh-7 cells to less than 5% at 50 μg/ml concentration compared to EAC, which decreased the colony formation ability of Huh-7 cells to about 58% at the same concentration (**Figures 3B,C**). These results confirmed that EACG has much better anticancer potential than EAC.

### Induction of G2/M Cell Cycle Arrest and Apoptosis by EACG

**FIGURE 4:**
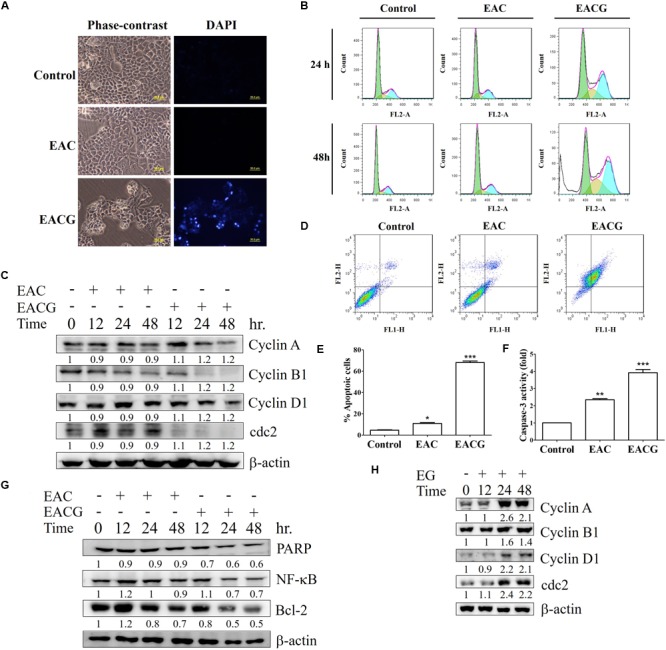
EACG induced G2/M arrest and cell apoptosis Huh-7 cells. **(A)** Huh-7 cells were treated with 50 μg/ml EAC or EACG for 48 h, the morphological changes were analyzed by fluorescence microscopy with DAPI staining. **(B)** Cell cycle analysis of Huh-7 cells treated with 50 μg/ml EAC or EACG in 10% FBS–containing DMEM for 24 and 48 h. The cells cycle was analyzed by flow cytometry after staining with propidium iodide. **(C)** Western blot analysis of Huh-7 cells treated with EAC or EACG for the indicated time points. Cell lysates were resolved and blotted with antibodies against cyclin A, cyclin B1, cyclin D1, cdc2, and β-actin. **(D)** Flow cytometric analysis of the apoptosis in Huh-7 cells treated with EAC or EACG at 50 μg/ml concentration after staining with fluorescein-conjugated Annexin V and propidium iodide. **(E)** Bar chart presenting the percent of cell numbers in the respective quadrants. **(F)** Caspase-3 activity in Huh-7 cells treated with EAC or EACG at 50 μg/ml concentration for 48 h. Columns, mean; bars, SD (*n* = 3). Significant difference between the control and experimental groups are marked with asterisks (^∗^*P* < 0.05; ^∗∗^*P* < 0.01; ^∗∗∗^*P* < 0.001). **(G)** The expression of apoptosis-associated protein was determined and blotted with antibodies against PARP, Bcl-2, Bax, MCL-1, Bcl-xL and β-actin. **(H)** Western blot analysis of Huh-7 cells treated with EG for the indicated time points. Cell lysates were resolved and blotted with antibodies against cyclin A, cyclin B1, cyclin D1, cdc2, and β-actin.

EAC and EACG-induced cell death in Huh-7 cells was mediated through apoptosis as indicated by the apoptotic bodies upon DAPI staining specially in EACG-treated cells (**Figure 4A**). In addition, the effect of EAC and EACG on cell cycle progression in Huh-7 cells were tested. Results revealed that EACG induced the accumulation of Huh-7 cells in G2/M phase and increased the population of sub-G1 cells in a dose-dependent manner (**Figure 4B**). As shown in **Table 3**, subG1 cells increased from 0.37% (control) to 17.78% (50 μg/ml EACG) after 36 h of treatment. Furthermore, EACG caused cells accumulation in G2/M phase form 17.64% (control) to 32.64% (50 μg/ml ACG). These results suggested the ability of EACG to induce G2/M phase cell cycle arrest in Huh-7 cells. Our previous study indicated that the regulation of cyclin A and cyclin B1 proteins expression was involved in G2/M arrest (Wu et al., 2015). In addition, the induction of G2/M arrest by gallic acid in TSGH-8301 cells was mediated through the downregulation of cyclin B1 and cdc2 expression (Ou et al., 2010). To understand the mechanism of EACG-induced effect on cell cycle progression, Western blot analysis was used to assess the changes in cyclin proteins expression. We found that EACG down-regulated cyclin A, cyclin B1, cyclin D1, and cdc2 expression (**Figure 4C**). These results could explain the cell cycle arrest that was detected by flow cytometry and the accumulation of high proportion of cells at G2/M. In order to evaluate the induction of cell apoptosis by EAC and EACG, the cells were determined by Annexin v and caspase-3 activity assay. Flow cytometric analysis of phosphatidylserine externalization as revealed by Annexin V staining showed that the treatment with EACG significantly induced apoptosis in Huh-7 cells (**Figure 4D**). For example, EACG at 50 μg/ml concentration increased the percentage of apoptotic cells to 65% while EAC at the same concentration increased it to about 10% relative to control cells (**Figure 4E**). Furthermore, we examined the effect of EAC or EACG on caspase-3 activity in Huh-7 cells using flow cytometric analysis. As shown in **Figure 4F**, exposure to EACG at 50 μg/ml concentration led to about 3.5-fold increase in caspase-3 activity compared to EAC at the same concentration, which caused a 1.8-fold increase. In addition, some proteins involved in cell apoptosis and signal regulation were determined such as Bcl-2, PARP and NF-κB. The downregulation of Bcl-2 and PARP cleavage were observed in apoptosis (Hung et al., 2008, 2011). NF-κB plays a key role in chronic liver disease, liver fibrosis and hepatocarcinogenesis (Hung et al., 2004; Reddy and Rao, 2006). In this study, the reduction of Bcl-2, PARP and NF-kB expression were induced by EACG treatment in Huh-7 cells (**Figure 4G**). In addition, to exclude the possibility that the observed effect on cell cycle was related to ginger alone, Huh-7 cells were treated with 50 μg/ml of the ethanolic extract of ginger (EG) for 0, 12, 24, and 48 h. Western blotting showed that EG alone at 50 mg/ml concentration increased the expression of cyclin A, cyclin B1, cyclin D1 and cdc2 especially after 24 and 48 h treatments (**Figure 4H**). The observed effect of EG alone on cell cycle proteins expression omitted the role EG on cell cycle arrest in EACG. More importantly, none of the known active ingredients of ginger such as gingerol, shogaol, paradols, or gingerdiols were significantly observed in EACG (**Table 1**).

**Table 3:** The effect of EAC and EACG on cell cycle distribution in Huh-7 cells.

		sub-G1	G0/G1	S	G2/M
24 h	Control	0.08 – 0.08	63.05 – 1.58	14.91 – 0.37	21.38 – 0.26
	EAC	0.56 – 0.27	63.52 – 0.53	14.41 – 0.76	19.84 – 0.87
	EACG	0.00 – 0.00	44.47 – 0.69*	17.95 – 0.24*	33.65 – 0.52**
36 h	Control	0.37 – 0.06	69.83 – 1.55	10.94 – 0.61	17.64 – 0.13
	EAC	0.30 – 0.23	66.36 – 2.07	11.87 – 0.68	20.62 – 0.70
	EACG	17.78 – 0.45***	27.75 – 1.63***	19.22 – 1.12**	32.64 – 0.91**

*Values are the cell population percent (%) ± SD (*n* = 3). Ethanoic extracts of *Antrodia cinnamomea* (EAC) alone or after the co-cultivation in presence of ginger (EACG). Significant difference between the control and experimental groups are marked with asterisks (^∗^*P* < 0.05; ^∗∗^*P* < 0.01; ^∗∗∗^*P* < 0.001).*

### Modulation of MAPK Signaling Pathways in ECAG-Treated Cells

**FIGURE 5:**
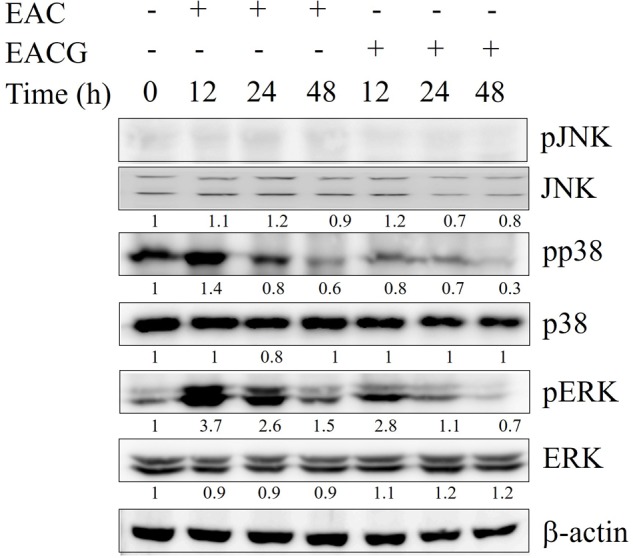
Modulation of MAPK signaling pathways by EAC and EACG. Western blot analysis of the time-dependent effects of EAC or EACG on the MAPK signaling pathways. Huh-7 cells were exposed to 50 μg/ml EAC or EACG in 10% FBS–supplemented DMEM for the indicated time points. The total lysates were resolved and blotted by antibodies against ERK1/2, p-ERK1/2, p-JNK, JNK, p38 and pp38.

Since MAPK families play an important role in cell cycle transition (Zhang and Liu, 2002), the effect of EAC and EACG on MAPK kinases signaling was determined. EACG treatment, significantly inhibited the phosphorylation of p38 and ERK in Huh-7 cells in a time-dependent manner (**Figure 5**). These results suggested that MAPK signaling pathways may have a role in the EACG-induced cellular stress.

### EACG Has Better Anticancer Activity Compared to AC Fruiting Body Extract

**FIGURE 6:**
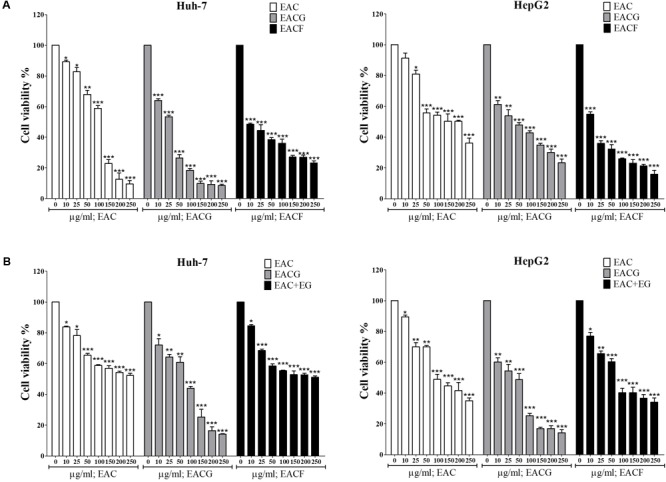
The effect of *A. cinnamomea* fruiting body (EACF) on cancer cell viability. **(A)** Huh-7 cells were exposed to EAC, EACG, and EACF at the indicated concentrations in 10% FBS-supplemented DMEM for 48 h, and cell viability was assessed by MTT assays. Points, mean; bars, SD (*n* = 6). **(B)** The effect of EAC combined with EG on Hhuh-7 and HepG2 cell viability. The cells were exposed to EAC, EACG or EAC with EG at 0, 10, 25, 50, 100, 150, 200, 250 μg/ml concentrations. The cell viability was determined by MTT assays. Significant difference between the control and experimental groups are marked with asterisks (^∗^*P* < 0.05; ^∗∗^*P* < 0.01; ^∗∗∗^*P* < 0.001).

A previous study indicated that extracts of *AC* fruiting body (EACF) have better anti-tumor activity than solid or liquid culture extract due to secondary metabolites. Therefore, we compared the anticancer activity of EAC, EACG and EACF in Huh-7 and HepG2 cells. The results showed that the growth rate of Huh-7 and HepG2 cells was inhibited by EAC, EACG and EACF. However, EACG was the most powerful inhibitor of cell growth in Huh-7 and HepG2 cells (**Figure 6A**). Furthermore, in order confirm the benefit of co-cultivation over the simple combination of ginger with *AC*, the ethanolic extracts of ginger (EG) was combined with *AC* and tested on Huh-7 and HepG2 cells by MTT assay. The result indicated that simple combination of EAC with EG did not significantly affect the action of EAC (**Figure 6B**). The normality distribution of cell survival results presented in **Figure 6** is available in Supplementary Materials

## Discussion

Hepatocellular carcinoma is a principal cause of cancer death worldwide and the long-term survival rates for liver cancer patients are one of the lowest for any cancer; therefore, new therapeutic agents are urgently needed (Omar et al., 2011). Many studies demonstrated that the extract of AC fruiting body, induces apoptosis in various tumor cell lines, such as lung cancer cells, leukemia cells, prostate, bladder cancer, colorectal cancer, and human cervical cancer cells (Lee et al., 2012; Peng et al., 2015; Chiu et al., 2016). These investigations demonstrated that the crude extract from fruiting assortments of AC specifically restrained the development of tumor cells with little impact on normal cells (Lee et al., 2012). The fruiting bodies of *AC* are usually collected from the wood of *Cinnamomum micranthum*. However, logging *Cinnamomum micranthum* trees has been totally prohibition by the Taiwanese government. Therefore, finding a new incubation method for *AC* is currently needed. In this study, we tried to establish a novel approach by the cocultivation of *AC* with other natural agents. In our previous study, we have reported the anticancer activity of ginger components such as 6-shogaol (Wu et al., 2015). Therefore, in the current study, we cultivated *AC* with or without fresh ginger then the ethanolic crude extracts from different cultivations were tested for their potential anticancer activity.

**FIGURE 7:**
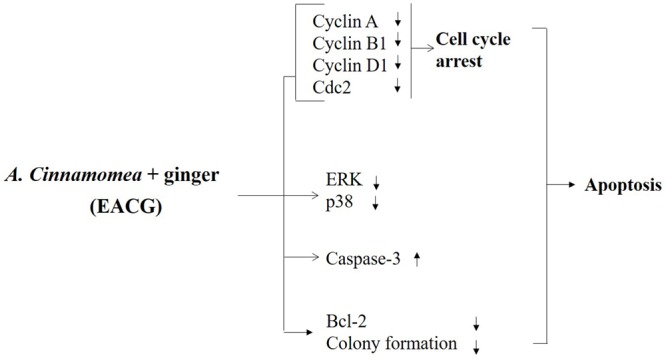
Proposed diagrams depicting effect of EACG on cell cycle, MAP kinases, and apoptosis signaling pathway. The modification of secondary metabolites of AC was induced by co-incubation with ginger. The interplay between these signaling networks at different cellular levels results in the ability of EACG to induce subG1/G2M arrest and apoptosis in Huh-7 cells.

The results showed that the anti-proliferative activity of EACG in Huh-7 and HepG2 cells was mediated through affecting multiple cancer cell signaling pathways. These cell lines were selected to have a different genetic background which usually results in different response to chemotherapeutic agents. For example, HepG2 cells express normal p53 while Huh-7 cells express mutated p53 and based on the role of p53 in the induction of apoptosis, we expected Huh-7 cells to be more resistant to the suggested treatments (Brito et al., 2012). The results confirmed our hypothesis since HepG2 cells were more sensitive to both EAC and EACG with IC_50_ of 51.93 and 8.35 μg/ml, respectively compared to Huh-7 cells which have IC_50_ values of 245.40 and 50.33 μg/ml to the same treatments indicating the role of the presence of wild-type 53 in the sensitivity toward these treatments. This pleiotropic anticancer mechanism in HCC was through the modulation of a wide spectrum of signaling effectors, including, NF-κB, MAPK kinases, caspase-3, and cytokines, leading to G2/M cell cycle arrest and apoptosis. The modulation of these signaling pathways could be indirect effects due to the induction of cancer cell stress and the initiation of apoptosis. The biological effect of natural agents is usually mediated through polypharmacology or the simultaneous modulation of different targets (Leonti and Casu, 2013; Omar et al., 2016a; Tolba et al., 2016). Thus, the unique ability of EACG to modulate these clinically relevant targets underlines EACG potential to be developed as a member in the therapeutic protocols of HCC (**Figure 7**).

Ginger is one of the most widely used dietary condiment which has many medicinal properties. There are many reported biologically active ingredients in ginger such as 6-gingerol, 8-gingerol, 10-gingerol, 6-shogaol, and 8-shogaol (Zick et al., 2008; Tao et al., 2009). These active compounds regulate many signal transduction pathways in cancer cell growth, angiogenesis and metastasis. For instance, 6-shogaol induced apoptosis in Huh-7 and HepG2 cancer cell lines through the activation of ROS (Wu et al., 2015). Ginger extracts also have been tested for antitumor activity in several *in vitro* cell lines, including leukemia (Omoregie et al., 2013), and gastric cancer (Tsuboi et al., 2014), prostate cancer (Brahmbhatt et al., 2013), ovarian cancer (Rhode et al., 2007), liver cancer (Wu et al., 2015), and lung carcinoma (Warin et al., 2014). In addition, the reported inhibition of MDA-MB-231 and HepG2 cancer cells invasion by 6-shogaol and 6-gingerol was mediated through the modulation of MMP-9 expression and NF-κB signal pathway (Ling et al., 2010; Weng et al., 2012). The safety of ginger components was reported before where ginger’s constituents at doses up to 2.0 g daily showed very low levels of toxicity in both animals and humans (Chrubasik et al., 2005). Therefore, ginger is a potential safe and effective candidate for the development of anticancer health food.

There are many reported trials to modulate and enhance the production active ingredients of *AC* through changing the cultivation conditions. For example, submerged fermentation of *AC* enhances the production of 4-acetylantroquinonol B, which inhibits hepatoma cell proliferation (Lin et al., 2010). Also, the cultivation of AC with orsellinic acid increased antroquinonol and 4-acetylantroquinonol synthesis (Chou et al., 2017). In addition, the cocultivation of *AC* with citrus peel extracts in liquid culture medium significantly induced the production of polyphenols and triterpenoids which serve as biologically active components of *AC* (Ma et al., 2014). Furthermore, many reports indicated that secondary metabolites in *AC* can be modified in submerged and solid culture (Lin and Sung, 2006; Chen et al., 2008; He et al., 2012). These secondary metabolites can be affected by light, nutrient, temperature and others which enable fungi to serve as biological factory of natural products and chemical modification (Khan et al., 2014). For example, the extract of *Aspergillus clavatus* incubated in 5′-azacytidine containing culture medium, exhibited antimicrobial activity against *Staphylococcus aureus* (Zutz et al., 2014). In addition, increased cytotoxic effect was observed in HepG2 and Caco2 cells upon the treatment with *Penicillium crustosum* co-incubated with trichostatin A (Zutz et al., 2014). Therefore, the modification of chemical compounds to enhance their biological activity can be completed using fungi as a tool via co-cultivation. In this study, we speculate that *AC* may be able to modify the composition of ginger active ingredients to boost its anticancer activity.

Previous studies indicated that the extract of *AC* fruit body entity which grows naturally on the wood of *Cinnamomum micranthum* in the wild has a better therapeutic effect (Zhao and Leung, 2010; Lin et al., 2011, 2017). However, the logging of *Cinnamomum micranthum* is already prohibited in Taiwan and the contamination by other fungi or bacteria is easily observed in the wood culture method. Therefore, the establishment of a novel fermentation method to replace the wood culture is important.

Considering the safety of *AC*, a randomized clinical trial indicated that the administration of *AC* for 8 weeks, did not cause any obvious adverse events nor abnormal laboratory findings throughout the study period (Chen et al., 2016). Another multiple-dose clinical study indicated the safety and efficacy of *AC* in human upon repeated administration in a dose up to 1250 mg/kg for 90 days (Hsiao et al., 2003). In animals, many studies indicated that the powder of *AC* fruiting body when given by oral gavage to rats at doses up to 3000 mg/kg/day for 90 consecutive days showed no systemic toxicity (Chen et al., 2011; Chang et al., 2013). Therefore, the safety of the novel extract presented in the current study provides a valuable developmental and therapeutic approach for the employment of *AC* as the safe adjuvant anticancer medication.

The identification of EACG components revealed the production of several secondary metabolites, which have anticancer activity such as antcin B, dehydrosulphurenic acid, 3β,15α-dihydroxy-lanosta-7,9(11),24-triene-21-oic acid and zhankuic acid C via different mechanisms. For example, zhankuic acid C and dehydrosulphurenic 3β,15α-dihydroxy-lanosta-7,9(11),24-triene-21-oic acid-induced apoptosis and activated caspase-3 activity in PC-3, prostate cancer cells (Lee et al., 2012). In addition, antcin B increased the percentage of sub-G1 cells and induced apoptotic cell death in HepG2 cells through ROS production (Hsieh et al., 2011). Moreover, zhankuic acid C induced apoptosis in colon, liver, breast and lung cancer cell lines (Yeh et al., 2009). The modulation of triterpenes production in AC via co-cultivation with other agents was reported before (Ma et al., 2014; Chou et al., 2017). The simple combination of EAC with EG failed to exhibit anticancer activity similar to EACG (**Figure 6B**). Therefore, we speculated that the significant anticancer activity of EACG is due to the new metabolites rather than the regular components of ginger extract. While these results shed the light on the anticancer activity of EACG, one of the major limitations of the current study is that we used the crude extract of EACG and the dominant compound in this extract remains to be further investigated. However, the use of the crude extract is a very common practice in the context of herbal medicines due to different levels of synergy between the active ingredients which contribute to the overall effect. Studies are ongoing to isolate the main active components in EACG and verifying their mechanism of action using HCC cell lines that ectopically express the main targets highlighted in the current study.

## Conclusion

The current study provides a novel approach for the optimization of *AC* cancer chemotherapeutic value in human HCC. The results highlighted an evidence that EACG is superior to EAC in targeting cancer cell survival and inducing apoptotic cell death in HCC. Further preclinical and animal studies are needed to support EACG formula as a potential candidate for HCC therapy.

## Author Contributions

S-YC, Y-RL, M-CH, HO, and J-HH conceived and designed the experiments. S-YC, Y-RL, M-CH, and HO performed the experiments. S-YC, Y-RL, M-CH, HO, and J-HH analyzed the data. S-YC, Y-RL, M-CH, HO, and J-HH contributed reagents, materials, and analysis tools. HO, Y-NT, C-YL, and J-HH wrote and revised the manuscript.

## Conflict of Interest Statement

The authors declare that the research was conducted in the absence of any commercial or financial relationships that could be construed as a potential conflict of interest. The reviewer EG and handling Editor declared their shared affiliation.

## Abbreviations

AC: *Antrodia cinnamomea*
DAPI: 4′,6-Diamidino-2-Phenylindole Dihydrochloride
EAC: ethanolic extracts of AC
EACF: ethanolic extract of AC fruiting body
EACG: ethanolic extracts of AC cocultivated with ginger
GADD 153: DNA damage-inducible gene 153
HCC: hepatocellular carcinoma
MAPK: mitogen-activated protein kinase
MTT: 3-(4,5-dimethylthiazol-2-yl)-2,5-diphenyltetrazoliumbromide
ROS: reactive oxygen species
