# Supplementary material for: Enhancing the Anticancer Activity of Antrodia cinnamomea in Hepatocellular Carcinoma Cells via Cocultivation With Ginger: The Impact on Cancer Cell Survival Pathways
Source: Front Pharmacol. 2018 Jul 18;9:780. doi: 10.3389/fphar.2018.00780 (PMC6058215; doi:10.3389/fphar.2018.00780)

Supplementary Material

1. The metabolite profiles of EACF, EAC and EACG as determined by HPLC/LC-MS/MS

EACF profiles:

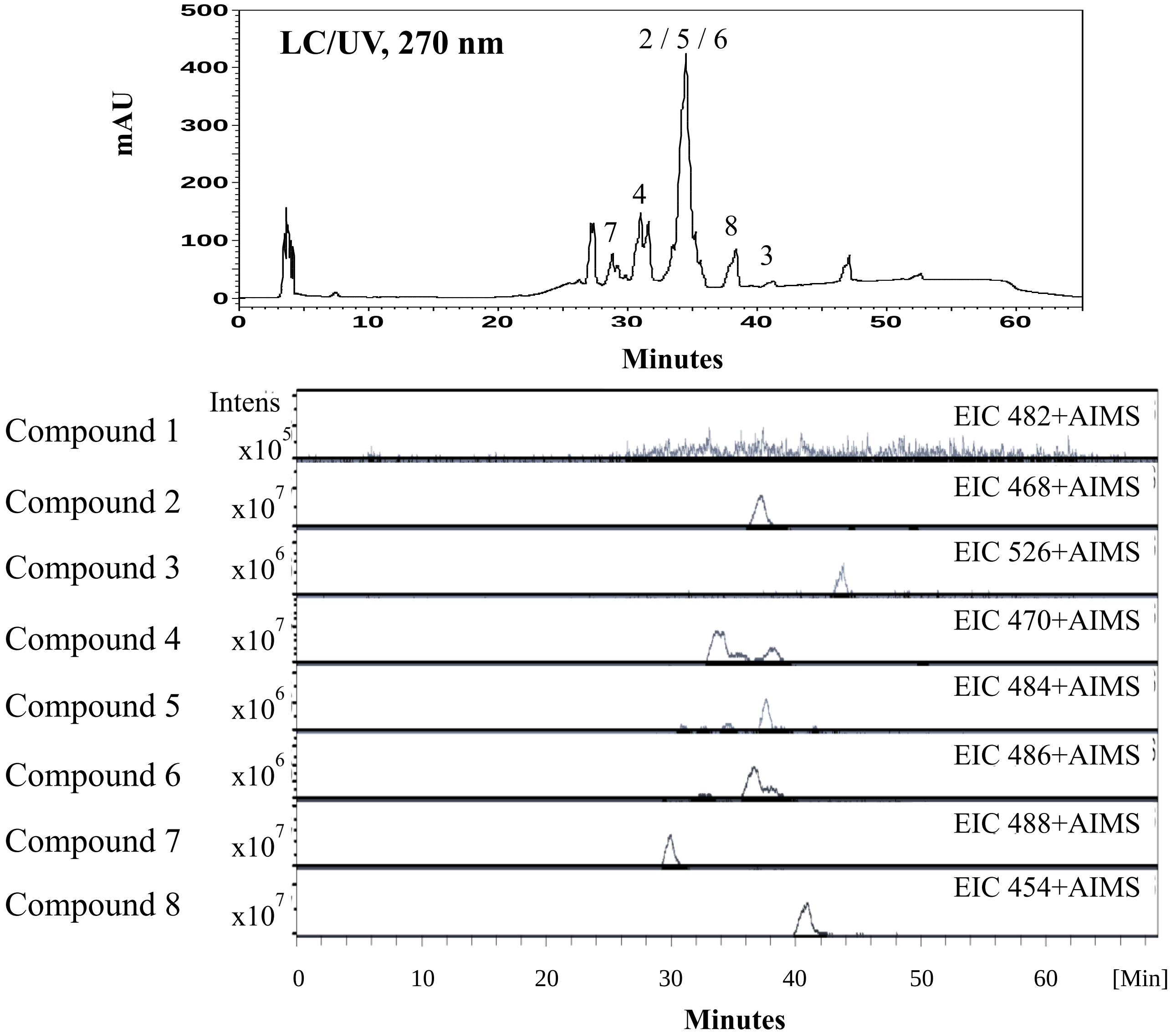

EAC profiles:

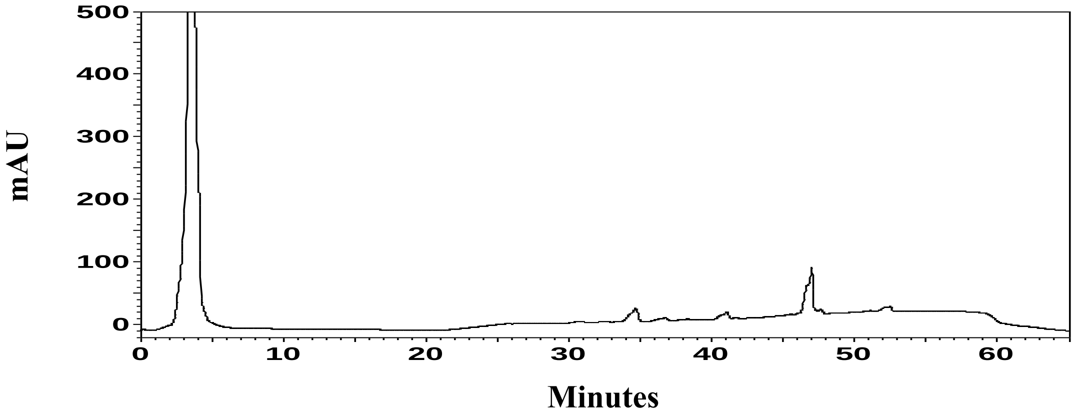

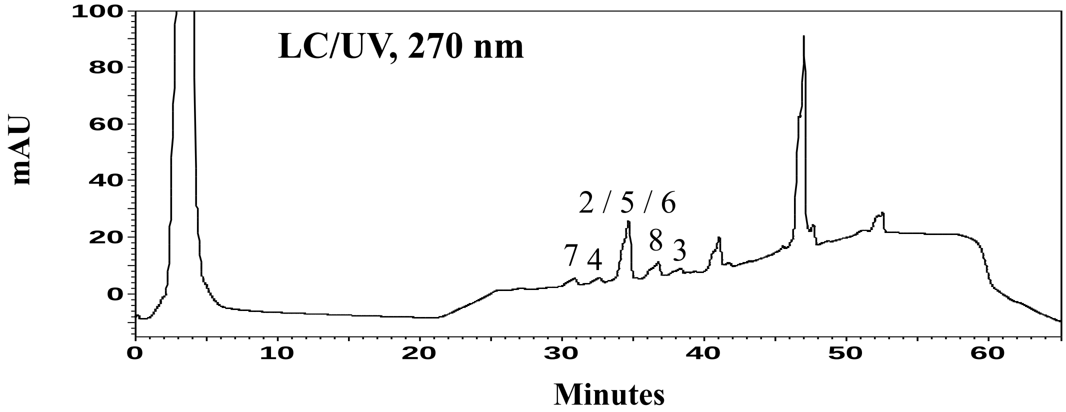

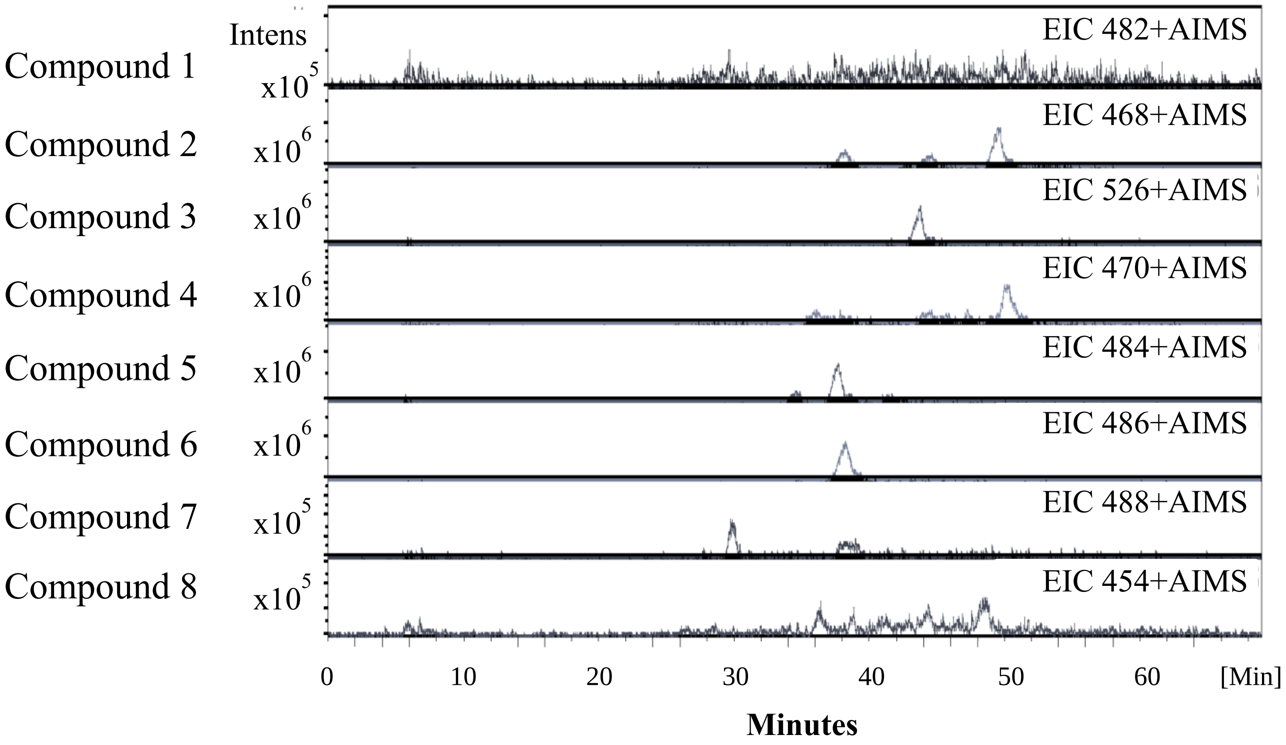

EACG profiles:

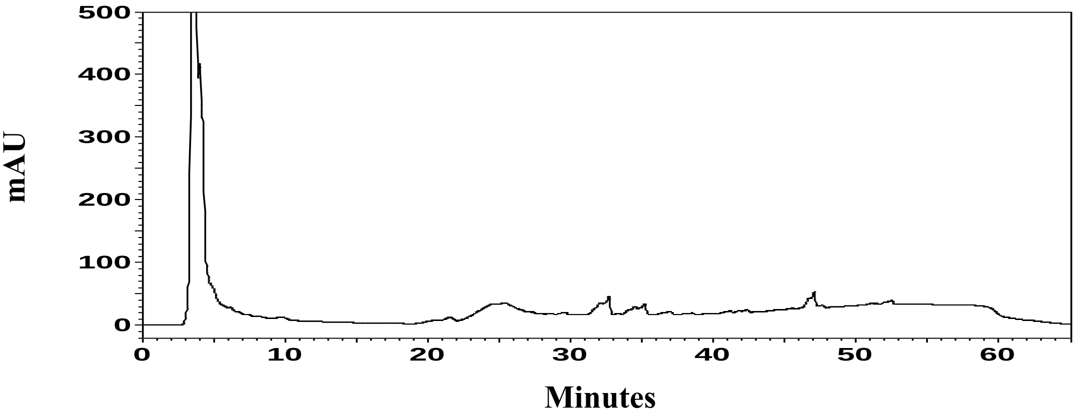

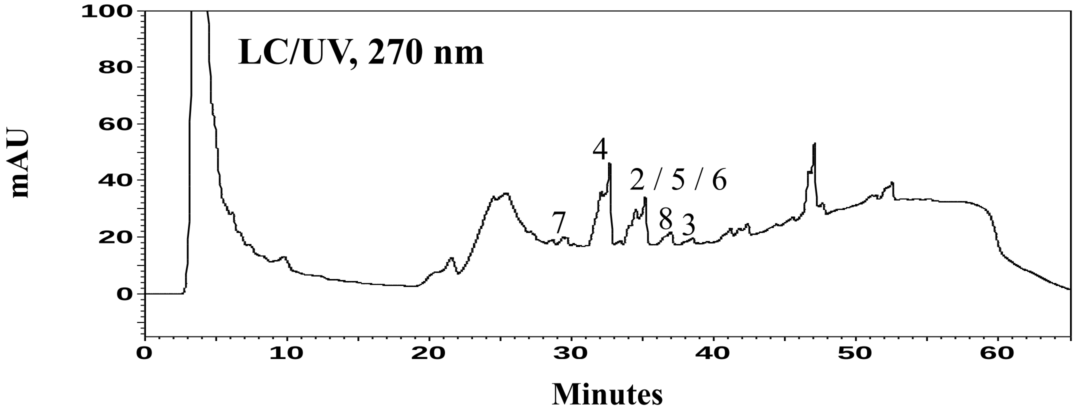

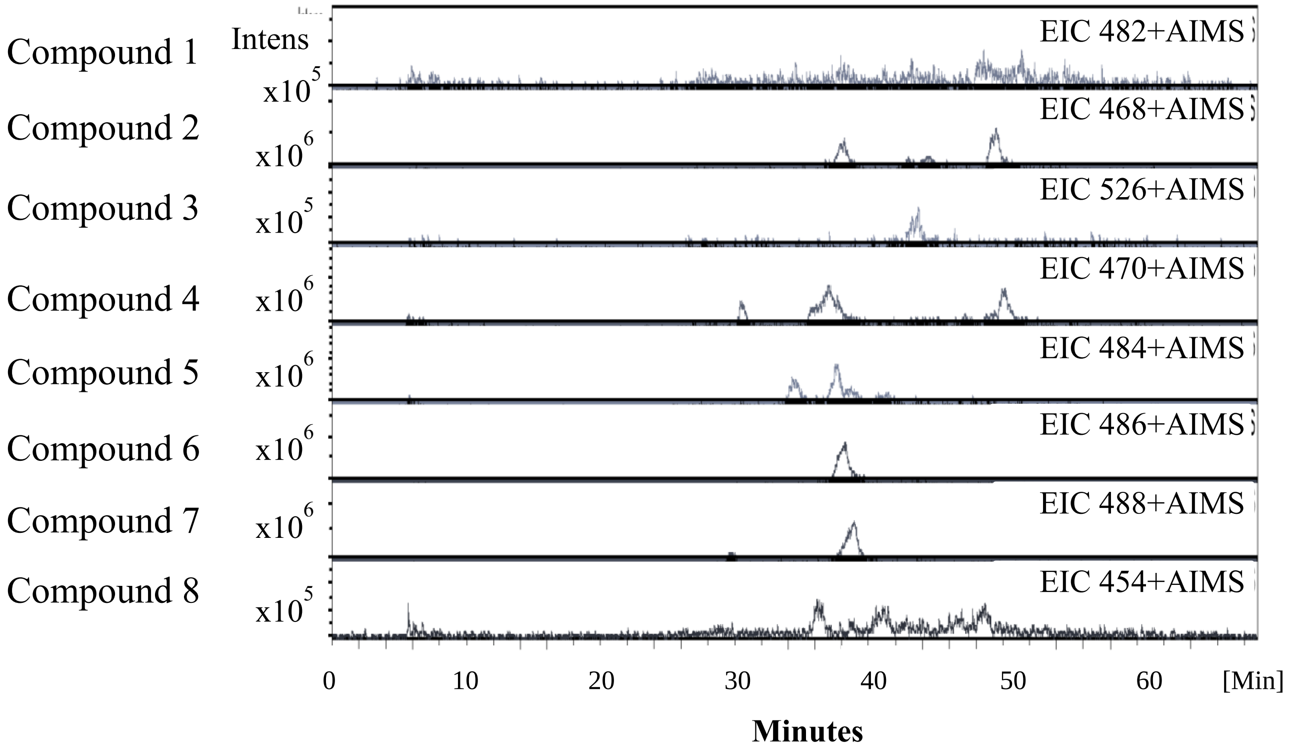

2. Peak area measurement of EACF, EAC and EACG as determined by HPLC/LC-MS/MS

No.	M.W.	RT(min) / peak area		
		EACF	EAC	EACG
1	482	-	56.3 / 45807096	-
2	468	37.2 / 1110639677	37.4 / 26290653<br>49.0 / 69841411<br>52.9 / 28626333	38.1 / 34282691<br>49.4 / 49679964
3	526	43.8 / 20897538	43.3 / 18429974	43.6 / 10011906
4	470	33.7 / 530371345	49.8 / 69841411	30.5 / 12568537<br>36.9 / 75492804<br>49.9 / 39265296
5	484	37.6 / 77466553	33.3 / 18429974<br>37.0 / 53354207	34.2 / 24078795<br>37.5 / 30690916
6	486	36.6 / 177827880	37.5 / 75112839	38.2 / 99318585
7	488	29.9 / 248615699	38.0 / 13035784	29.7 / 7712639<br>38.9 / 77542852
8	454	40.8 / 807620525	-	36.1 / 12060920<br>40.5 / 10684017<br>48.6 / 8644112

3. Normality distribution of cell survival data

Figure 2

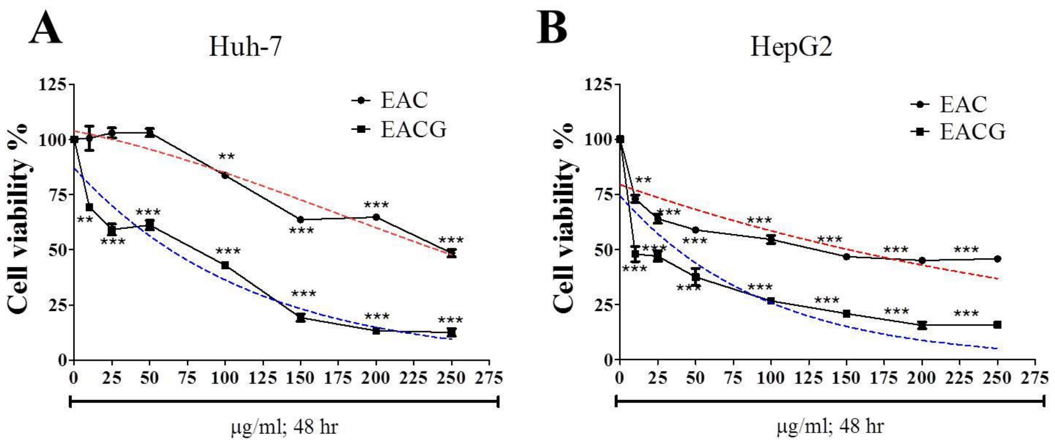

Figure 6

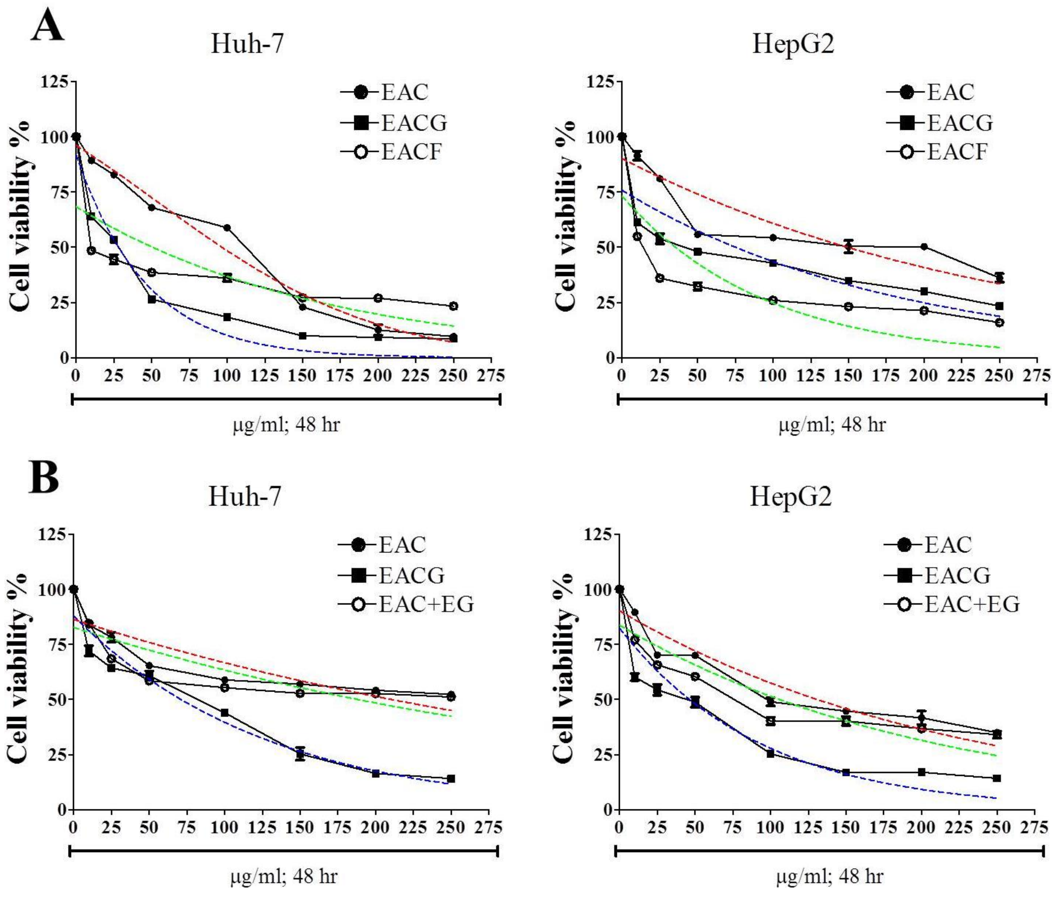

Supplement: Supplementary file 1 [file Data_Sheet_1.PDF]
